# Acute taVNS modulates lateralized hypoactivity in the right cerebral hemisphere in individuals with subjective cognitive decline

**DOI:** 10.3389/fnins.2026.1884933

**Published:** 2026-08-25

**Authors:** Xiaocheng Li, Jiazhu Huang, Liyuan Zhou, Bo Liu, Kaixuan Zhou, Shihuan Lin, Haixia Qin, Ruijing Sun, Yichen Wei, Ying Liu, Jinli Huang, Demao Deng, Lingyan Liang

**Affiliations:** 1 Department of Radiology, The People's Hospital of Guangxi Zhuang Autonomous Region, Guangxi Academy of Medical Sciences, Nanning, Guangxi, China; 2 Department of Radiology, The First People's Hospital of Fangchenggang, Fangchenggang, Guangxi, China; 3 Department of Neurology, The People’s Hospital of Guangxi Zhuang Autonomous Region, Guangxi Academy of Medical Sciences, Nanning, Guangxi, China; 4 Department of Radiology, Hainan General Hospital (Hainan Affiliated Hospital of Hainan Medical University), Haikou, Hainan, China

**Keywords:** Alzheimer’s disease, amplitude of low frequency fluctuation, right-lateralization, subjective cognitive decline, transcutaneous auricular vagus nerve stimulation

## Abstract

**Background:**

Subjective cognitive decline (SCD) is considered a preclinical stage of Alzheimer’s disease and represents a critical window for early intervention. Transcutaneous auricular vagus nerve stimulation (taVNS) can modulate cognition-related brain networks. However, its effects in SCD remain unclear.

**Purpose:**

This study aimed to investigate the immediate neural responses of the brain to taVNS at different frequencies in individuals with SCD.

**Methodology:**

Fifty-one individuals with SCD and forty healthy controls (HCs) were enrolled. Individuals with SCD underwent acute taVNS at 1 Hz and 20 Hz, as well as sham stimulation. The amplitude of low-frequency fluctuation (ALFF) was used to assess regional brain activity and the immediate effects of different taVNS frequencies in SCD.

**Results:**

Forty-nine SCD and 39 HC were included. Compared with HCs, SCD exhibited reduced ALFF values in the right inferior frontal gyrus (opercular part), right inferior temporal gyrus, right middle temporal gyrus, and right superior frontal gyrus, and these reductions were associated with subjective cognitive complaints and some standard neuropsychological measures. In SCD, 1 Hz taVNS increased ALFF values in the right fusiform, lingual, inferior occipital, middle temporal, and inferior temporal gyri, and decreased ALFF in the left superior and middle frontal gyri. Similar patterns were observed with 20 Hz taVNS, with additional modulation of the left insula.

**Conclusion:**

SCD showed right-frontotemporal hypoactivity that may reflect an underlying pathophysiological mechanism. Both 1 Hz and 20 Hz taVNS can modulate the intrinsic neural activity within multiple cognitive-related brain networks SCD. Long-term intervention and comprehensive cognitive testing are required to verify persistent clinical benefits.

## 1 Introduction

Alzheimer’s disease (AD) is a neurodegenerative disorder characterized by insidious onset, progressive cognitive decline, and deterioration in daily functioning. It is currently the fifth leading cause of death in both urban and rural populations in China (Ren et al., 2022), with incidence and mortality rates exceeding global averages. Although anti-amyloid therapies such as leqembi and donanemab have recently been approved by the FDA for patients with mild cognitive impairment (MCI) and mild AD dementia, their clinical benefits remain modest, long-term efficacy is uncertain, and safety concerns persist (Walsh et al., 2024). Limited treatment efficacy at the MCI stage may reflect established and potentially irreversible neuronal damage (Jessen et al., 2014), highlighting the need to shift intervention to earlier stages of the disease continuum. Subjective cognitive decline (SCD) is widely regarded as a preclinical stage of AD (Jessen et al., 2014; Jessen et al., 2020). It is associated with brain network alterations similar to those observed in AD, as well as an increased risk of subsequent cognitive decline and dementia (Jessen et al., 2014; Jack et al., 2018; Slot et al., 2019). Therefore, SCD represents a potential window for early identification and intervention. However, no evidence-based treatments are currently recommended for SCD.

Transcutaneous auricular vagus nerve stimulation (taVNS) is a noninvasive neuromodulation therapy derived from traditional invasive vagus nerve stimulation and grounded in vagal neuroanatomy. TaVNS has shown significant efficacy in various neuropsychiatric disorders and has been approved by the FDA for the treatment of treatment-resistant epilepsy, depression, and headaches (Yuan and Silberstein, 2016; Mwamburi et al., 2017). Mechanistically, taVNS-induced auricular vagal afferent signals may be integrated and relayed through the nucleus tractus solitarius, subsequently engaging multiple neuromodulatory systems, including the locus coeruleus–noradrenergic (LC–NE), cholinergic, dopaminergic, and serotonergic systems (Urbin et al., 2021). The LC projects to various brain regions, including the medial prefrontal cortex, the limbic system, thalamus, anterior cingulate cortex, which are involved in cognition (Jin et al., 2026). The cholinergic system primarily originates from the basal forebrain nuclei and sends widespread projections to the hippocampus, entorhinal cortex, and cingulate cortex (Jin et al., 2026). Diminished nicotinic acetylcholine receptors activity drives DMN dysregulation across MCI and AD (Zhang et al., 2010). Neurons in the nucleus raphe medius and the nucleus raphe dorsalis project abundant serotonergic fibers to the hippocampus and cortex. Selective 5-HT reuptake inhibitors boost DMN connectivity with the precuneus and posterior cingulate cortex (Klaassens et al., 2019). DA neurons are chiefly situated in midbrain ventral tegmental area and substantia nigra pars compacta, and projects to the prefrontal cortex, nucleus accumbens, hippocampus and dorsal striatum. Abnormal dopaminergic regulation within the substantia nigra and striatum contributes to deficits in visuospatial and executive performance in AD patients (Kang et al., 2023). These distributed neuromodulatory effects may collectively influence cognitive-related brain network activity, supporting taVNS as a potential intervention strategy for SCD. However, the optimal stimulation parameters for taVNS are yet to be established.

Stimulation frequency is a crucial parameter for taVNS, with different frequencies producing distinct physiological and clinical responses. For example, compared with 20 Hz stimulation, 1 Hz taVNS more strongly modulated functional connectivity in the periaqueductal gray matter in patients with migraine (Cao et al., 2021). In contrast, in drug-resistant epilepsy, 25 Hz stimulation was associated with a greater, although not statistically significant, reduction in seizure frequency compared with the 1 Hz (Bauer et al., 2016). Therefore, it is imperative to systematically evaluate the frequency-specific effects of taVNS. A recent scoping review summarized that 20 Hz and 25 Hz are the two most prevalent pulse frequencies adopted for taVNS clinical practice (Gerges et al., 2024). Nevertheless, most VNS studies focusing on patients with AD and MCI have adopted 20 Hz stimulation (Murphy et al., 2023; Wang et al., 2022; Sjögren et al., 2002; Merrill et al., 2006). More importantly, the physiological firing range of the locus coeruleus noradrenergic neurons is confined to 10–20 Hz; this phasic burst firing generates sharp NE surges supporting synaptic remodeling and memory stabilization (Ludwig et al., 2021; Aston-Jones and Cohen, 2005). Taken together, in the present study, 1 Hz and 20 Hz were selected to represent low- and high-frequency stimulation, respectively, with consideration of safety and prior evidence.

Resting-state functional MRI (rs-fMRI) enables the assessment of spontaneous brain activity and functional connectivity between regions by measuring intrinsic blood oxygen level-dependent (BOLD) low-frequency signal fluctuations. This approach provides insights into intrinsic brain networks, cognitive processes, and disease-related pathophysiology (Zhang and Raichle, 2010). The amplitude of low-frequency fluctuation (ALFF) is a widely used rs-fMRI metric that quantifies the intensity of local spontaneous neural activity within the 0.01 Hz to 0.1 Hz frequency band during the resting state. ALFF demonstrates good sensitivity for detecting intergroup differences and offers an optimal balance between test reliability and reproducibility (Zang et al., 2007; Chen et al., 2018). Therefore, it is well-suited to characterize local brain activity changes associated with SCD and to evaluate the neural effects of taVNS.

The primary objectives of this study were to characterize regional brain activity in individuals with SCD using ALFF and to investigate the immediate modulatory effects of low-frequency (1 Hz) and high-frequency (20 Hz) taVNS. We hypothesized that SCD is associated with altered intrinsic brain activity and which could be modulated by acute taVNS.

## 2 Materials and methods

This study was conducted at the People’s Hospital of Guangxi Zhuang Autonomous Region and was approved by the Ethics Committee of Guangxi Zhuang Autonomous Region People’s Hospital (KY-GZR-2021-104). It has been registered in the Chinese Clinical Trial Registry (ChiCTR2200058427) on April 9, 2022. All procedures were conducted in accordance with the principles outlined in the Declaration of Helsinki. Written informed consent was obtained from all participants or their legally authorized representatives at the time of enrollment.

### 2.1 Study design

This study employed a randomized, participant-blinded crossover design. At baseline, all participants, including individuals with SCD and healthy controls (HCs), underwent rs-fMRI and T1-weighted imaging (T1WI). In the SCD group, additional rs-fMRI data were acquired during three stimulation conditions: 20 Hz taVNS, 1 Hz taVNS, and sham taVNS (staVNS). The order of stimulation conditions was randomized to minimize order effects. To reduce potential carryover and sensitization effects, each intervention was separated by a washout period of at least 24 h, and all three conditions were completed within 1 week.

### 2.2 Participants

A total of 51 individuals with SCD and 40 HCs were recruited from local communities and outpatient clinics between December 2022 and December 2023.

Inclusion criteria were as follows: (1) age 55–75 years; (2) right-handedness; (3) normal performance on standard neuropsychological tests used to screen for MCI and prodromal AD; (4) no hearing, visual, or language communication impairments that could interfere with study assessments. Exclusion criteria included: (1) diagnosis of MCI or AD; (2) central nervous system conditions, such as brain tumors, Parkinson’s disease, encephalitis, epilepsy, or significant white matter lesions (modified Fazekas score ≥2); (3) systemic conditions associated with cognitive impairment, such as thyroid dysfunction, severe anemia, hepatic encephalopathy, syphilis, and HIV infection; (4) psychiatric disorders (e.g., depression, anxiety) or substance abuse; (5) use of medications that may affect cognitive function or cause failure of vital organs (heart, brain, kidney) within the specified washout period; (6) relevant cardiovascular and cerebrovascular risk factors, including hypertension, dyslipidemia, or diabetes mellitus. (7) left ear deformity or recent trauma; (8) contraindications to MRI, including non-removable metallic implants or claustrophobia. Participants were classified as having SCD3 if they reported a subjective decline in memory within the preceding 5 years, either self-reported or confirmed by a reliable informant, accompanied by concern and a perception of worse cognitive performance compared with age-matched peers. Participants who did not meet these criteria were assigned to the HC group.

The neuropsychological tests included the Subjective Cognitive Decline-Questionnaire (SCD-Q), Mini-Mental State Examination (MMSE), and Montreal Cognitive Assessment-Basic (MoCA-B) to evaluate global cognitive function. Domain-specific cognitive functions were assessed using the Auditory Verbal Learning Test (AVLT) for memory, the Shape Trail Test (STT) for executive function, and the Animal Fluency Test (AFT) and Boston Naming Test (BNT) for language. The full battery of neuropsychological assessments was administered in all both groups only at baseline.

### 2.3 MRI data acquisition

MRI data were acquired using a 3.0 T Siemens MAGNETOM Vida scanner (Siemens Healthineers) equipped with a 64-channel head coil. taVNS was delivered via a circular electrode attached to the left ear, connected to an external stimulator through an extended, electromagnetically shielded cable, allowing simultaneous stimulation during MRI acquisition. During rs-fMRI scanning, participants’ heads were stabilized with foam padding to minimize motion artifacts. Participants were instructed to remain awake, keep their eyes open, and wear earplugs to reduce scanner noise. The specific scan sequences and parameters were as follows: T1-weighted image: 3D MPRAGE sequence, TR = 2000 ms, TE = 2.04 ms, FOV = 256 mm × 256 mm, slice thickness = 1 mm, resolution = 256 × 256, flip angle = 7°. rs-fMRI: EPI sequence, TR = 2000 ms, TE = 30 ms, FOV = 220 mm × 220 mm, slice thickness = 2.5 mm, resolution = 88 × 88, flip angle = 90°.

### 2.4 Transcutaneous auricular vagus nerve stimulation protocol

taVNS was delivered using a TENS-200 stimulator. Stimulation parameters were as follows: frequency 1 Hz or 20 Hz; pulse width 250 μs; current intensity ≤ 3.0 mA, determined by individual tolerance of the patients and defined as the maximum tolerable level that elicited a clear but comfortable auricular sensation, such as a tingling or pulsation; and duration 8 min. The cymba conchae of the left ear was selected as the active stimulation site, as it is capable of eliciting robust brain responses while minimizing potential cardiac effects. The outer margin of the left auricle (upper scapha) was used as the sham stimulation site, this region lacks vagal innervation and is commonly used as the control site in taVNS studies (Peuker and Filler, 2002).

### 2.5 Image data processing

The fMRI data preprocessing was performed using the Data Processing & Analysis for Brain Imaging (DPABI, version 6.0) implemented in MATLAB. The preprocessing steps were as follows: (1) Format Conversion: Functional and structural images were converted from DICOM to NIFTI format. (2) Initial Signal Removal: The first 10 time points were discarded to reduce the impact of initial signal instability. (3) Slice Timing Correction: Slice timing delays among the remaining 230 images were corrected and realigned to the last slice. (4) Head Motion Correction: Head motion correction was performed. (5) T1 Segmentation: T1-weighted images were segmented. (6) Covariate Regression: Covariates, including 24 head motion parameters, cerebrospinal fluid, and white matter signals, were regressed out, and linear drift was removed. (7) Normalization: All head images were normalized to Montreal Neurological Institute (MNI) standard space using the DARTEL template and resampled to a voxel size of 3 mm × 3 mm × 3 mm. (8) Smoothing: Spatial smoothing was applied using a 6 mm Full Width at Half Maximum Gaussian kernel to increase signal-to-noise ratio and reduce registration errors.

The voxel-based Amplitude of Low-Frequency Fluctuations (ALFF) values were calculated from the DPABI-preprocessed fMRI data as follows: (1) Bandpass Filtering: A bandpass filter (0.01–0.1 Hz) was applied to reduce high-frequency physiological noise and low-frequency drift. (2) Fourier Transform: Fast Fourier Transform was used to convert the filtered time series into the frequency domain and obtain the power spectrum. (3) Square Root Calculation: The square root of the power at each frequency was computed, and the mean square root within the 0.01–0.1 Hz range was calculated as the ALFF value. (4) Normalization: ALFF values were standardized.

### 2.6 Statistical analysis

Demographic and neuropsychological test data were analyzed using SPSS version 20.0. Categorical variables (e.g., sex) were summarized as frequencies and compared using chi-square tests. Continuous variables were assessed for normality using the Shapiro–Wilk test. Normally distributed variables were compared using independent-samples *t*-tests, while non-normally distributed variables were compared using the Mann–Whitney U test. The significance level was set at *p* < 0.05.

Between-group differences in ALFF values (SCD vs. HCs) were assessed using two-sample t-tests in DPABI, with sex included as a covariate. Statistical significance was determined using Gaussian random field (GRF) correction, with a voxel-level threshold of *p* < 0.001 and a cluster-level threshold of *p* < 0.05. Within the SCD group, differences across conditions (baseline, 1 Hz taVNS, 20 Hz taVNS, and staVNS) were evaluated using one-way repeated-measures analysis of variance (ANOVA) in SPM12. Multiple comparisons were controlled using false discovery rate (FDR) correction at *p* < 0.05. *Post hoc* paired *t*-tests were subsequently conducted in DPABI to identify condition-specific activation differences, with GRF correction applied (voxel-level *p* < 0.001, cluster-level *p* < 0.05, two-tailed).

Mean ALFF values were extracted from brain regions showing significant between-group differences after adjustment for sex. Associations between regional ALFF values and neuropsychological test scores were assessed using partial Spearman correlation analyses controlling for sex. Statistical significance was set at *p* < 0.05.

Exploratory ROI based analyses were conducted to directly compare ALFF responses to 1 Hz and 20 Hz taVNS. ROI masks were generated from brain regions showing (1) significant baseline ALFF differences between the SCD and HC groups and (2) significant condition effects identified by one-way repeated-measures F-tests across stimulation conditions. Mean ALFF values were extracted from the resulting five data derived ROIs under the resting state, 1 Hz taVNS, and 20 Hz taVNS conditions. Stimulation induced ALFF responses were calculated as follows:

$${\text{Effect}}_{1\;\mathrm{Hz}}={\text{ALFF}}_{1\;\mathrm{Hz}\;\text{taVNS}}-{\text{ALFF}}_{\text{Rest}}$$

$${\text{Effect}}_{20\;\mathrm{Hz}}={\text{ALFF}}_{20\;\mathrm{Hz}\;\text{taVNS}}-{\text{ALFF}}_{\text{Rest}}$$

The responses to 1 Hz and 20 Hz taVNS were directly compared within each ROI using paired t tests. Bonferroni correction was applied across the five ROI comparisons, with statistical significance defined as an uncorrected *p* < 0.010.

## 3 Results

### 3.1 Demographic and clinical characteristics

**Figure 1:**
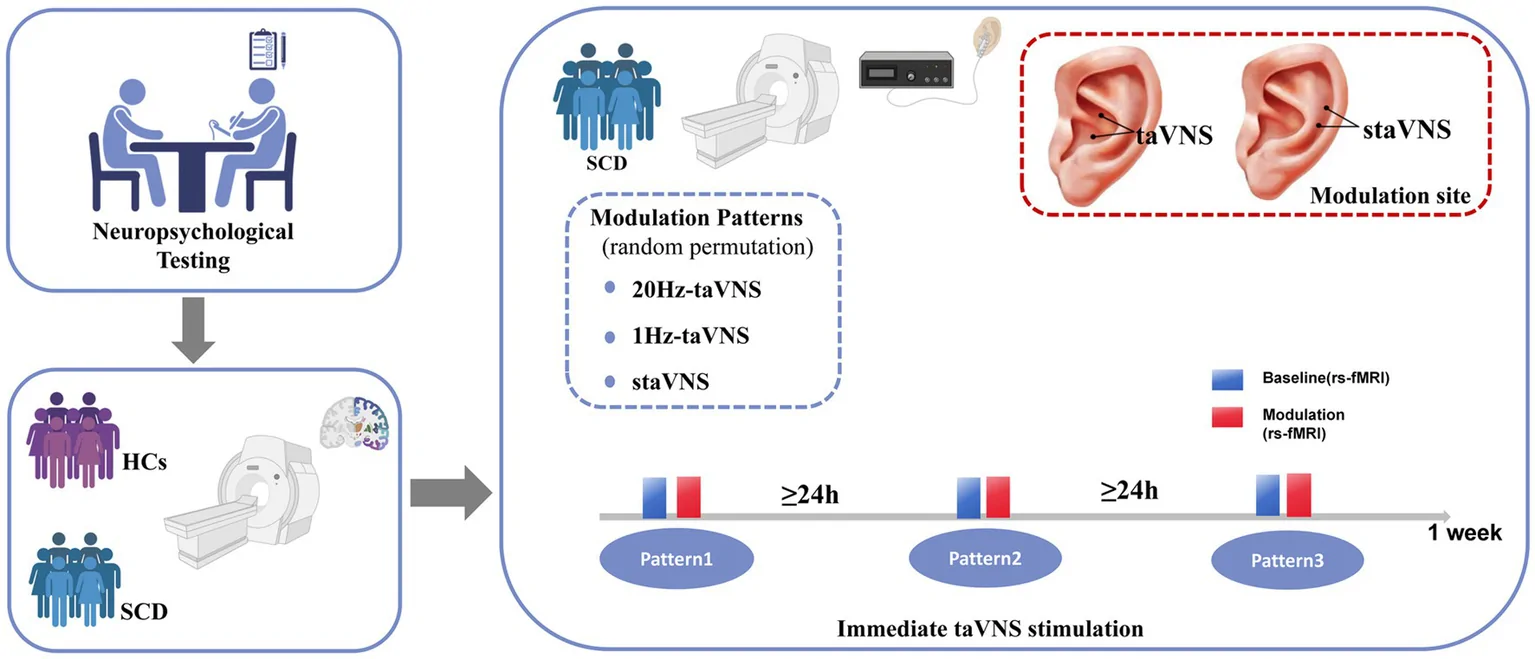
Study design flowchart. Participants completed neuropsychological assessments. At baseline, both SCD and HC groups underwent T1WI and rs-fMRI. The SCD group then received randomized 1 Hz taVNS, 20 Hz taVNS, and sham stimulation during simultaneous rs-fMRI, with ³24 h washout between sessions. All conditions were completed within one week.

A schematic overview of the study design is presented in Figure 1. A total of 49 individuals with SCD and 39 HCs were included in the final analysis after excluding two SCD participants and one HC due to incomplete imaging data or excessive artifacts. There were no significant differences between groups in age or years of education (*p* > 0.05). Sex distribution showed a numerical imbalance between groups, although the difference did not reach statistical significance (*p* = 0.063). Compared with HCs, the SCD group had lower scores on the MMSE and delayed recall measures of the AVLT (N5, N7) (all *p* < 0.05). SCD-Q scores in the SCD group were significantly higher than those in HCs (*p* < 0.001). No significant between-group differences were observed in MoCA-B, immediate recall measures of the AVLT, STT-A and STT-B, AFT, and BNT scores (all *p* > 0.05) (Table 1).

**Table 1:** Demographic and clinical neuropsychological data.

Characteristics		SCD (*n* = 49)	HCs (*n* = 39)	*Z/c* ^2^	*p-*value
Sex	M	9/49 (18.4%)	14/39 (35.9%)	3.457	0.063
	F	40/49 (81.6%)	25/39 (64.1%)		
Age (years)		60 (57, 65)	59 (56, 65)	−0.372	0.710
Education (years)		12 (9, 15)	12 (9, 15)	−1.198	0.231
Neuropsychological testing					
SCD-Q		5.0 (3.5, 6.0)	1.0 (0.0, 2.5)	−6.448	<0*.001*
MMSE		29.0 (28.0, 29.0)	29.0 (29.0, 30.0)	−2.684	0.007
MoCA-B		25.0 (23.0, 27.0)	26.0 (24.0, 27.0)	−0.664	0.507
AVLT-N5		5.0 (3.5, 6.0)	6.0 (5.0, 7.0)	−2.120	0.034
AVLT-N7		21.0 (19.5, 22.5)	23.0 (20.0, 23.0)	−2.198	0.028
STT-A		61.0 (45.0, 73.5)	60.0 (50.0, 91.0)	−0.794	0.427
STT-B		146.0 (107.0, 176.0)	147.0 (100.0, 180.0)	−0.424	0.671
AFT		16.0 (13.0, 18.0)	17.0 (14.0, 20.0)	−1.024	0.306
BNT		26.0 (22.5, 29.0)	26.0 (24.0, 28.0)	−0.042	0.966

SCD, subjective cognitive decline; HC, healthy control. MMSE, Mini-Mental State Examination; MoCA-B, Montreal Cognitive Assessment-Basic; SCD-Q, Subjective Cognitive Decline-Questionnaire; STT, Shape Trail Test; AVLT-N5, Audio Verbal Learning Test: 20-min long-term delayed recall; AVLT-N7, Auditory Verbal Learning Test: recognition; AFT, Animal Fluency Test; BNT, Boston Naming Test. p-values were calculated with the Mann–Whitney U-test.

### 3.2 ALFF contrasts

**Figure 2:**
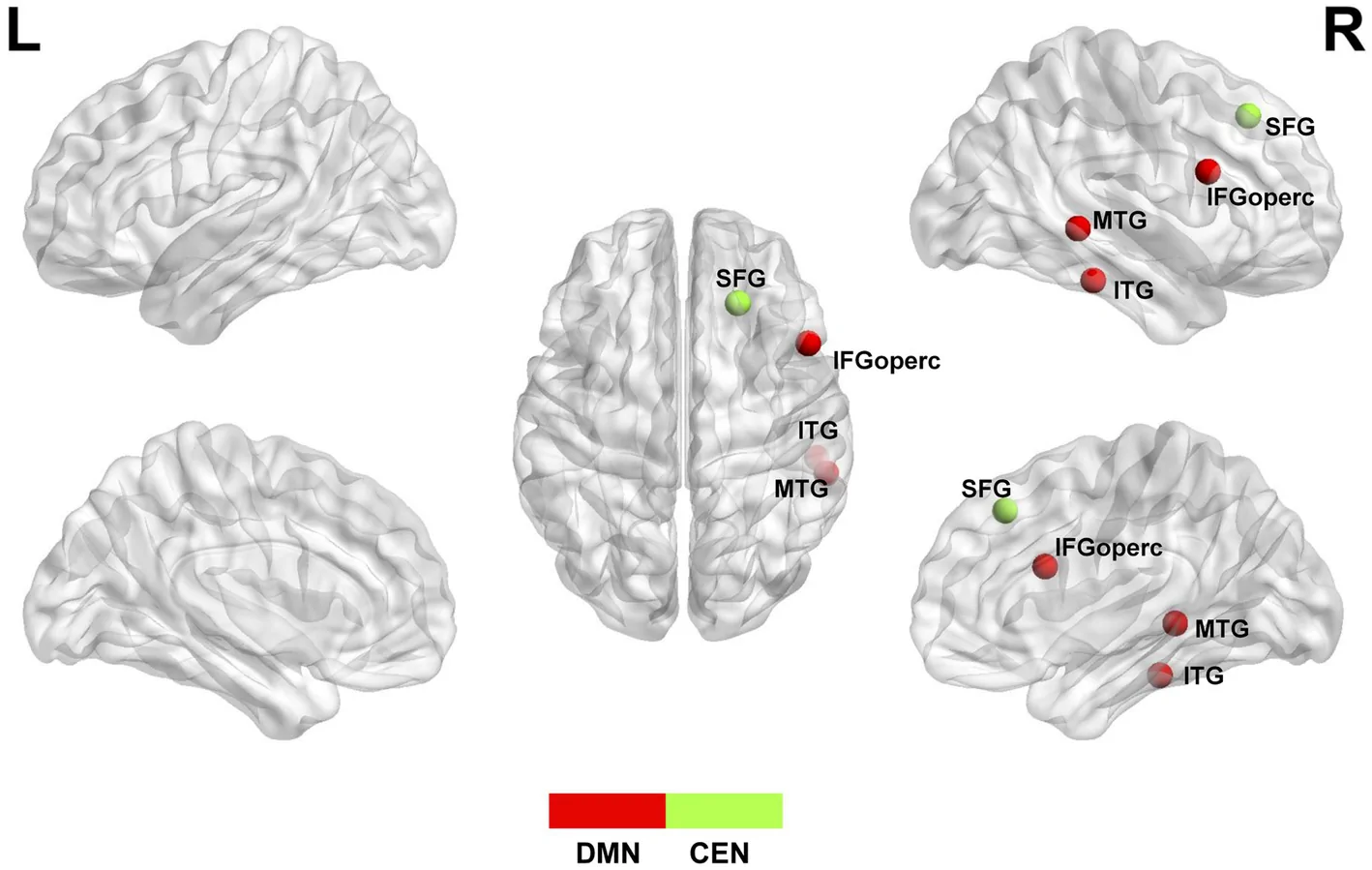
Changes in regional brain activity in SCD. SCD showed right-lateralized reductions in brain activity. ITG, inferior temporal gyrus; SFG, superior frontal gyrus; IFGoperc, inferior frontal gyrus, opercular part; MTG, middle temporal gyrus.

Compared with HCs, individuals with SCD exhibited reduced ALFF values in the right inferior frontal gyrus (opercular part) (Frontal_Inf_Oper_R), right inferior temporal gyrus (Temporal_Inf_R), right middle temporal gyrus (Temporal_Mid_R), and right superior frontal gyrus (Frontal_Sup_R) (voxel level *p* < 0.001, cluster level *p* < 0.05, GRF correction, two-tailed) (Table 2; Figure 2).

**Table 2:** Changes of regional brain activity in SCD at baseline.

	Brain regions	Cluster size	*t*-value	Peak MNI			ALFF value
				X	Y	Z	
Cluster1	Frontal_Inf_Oper_R	27	−5.7559	48	15	15	Decreased
Cluster2	Temporal_Inf_R	28	−4.6723	51	−51	−12	Decreased
Cluster3	Temporal_Mid_R	19	−4.478	42	−57	9	Decreased
Cluster4	Frontal_Sup_R	18	−4.6833	24	54	6	Decreased

SCD, subjective cognitive decline; HC, healthy control; MNI, Montreal Neurological Institute; X, Y, Z, indicate the coordinates according to the MNI. ALFF, amplitude of low-frequency fluctuation. Sex was included as a covariate in the between-group analysis. Statistical significance was defined as voxel-level *p* < 0.001, cluster-level *p* < 0.05, with GRF correction.

**Figure 3:**
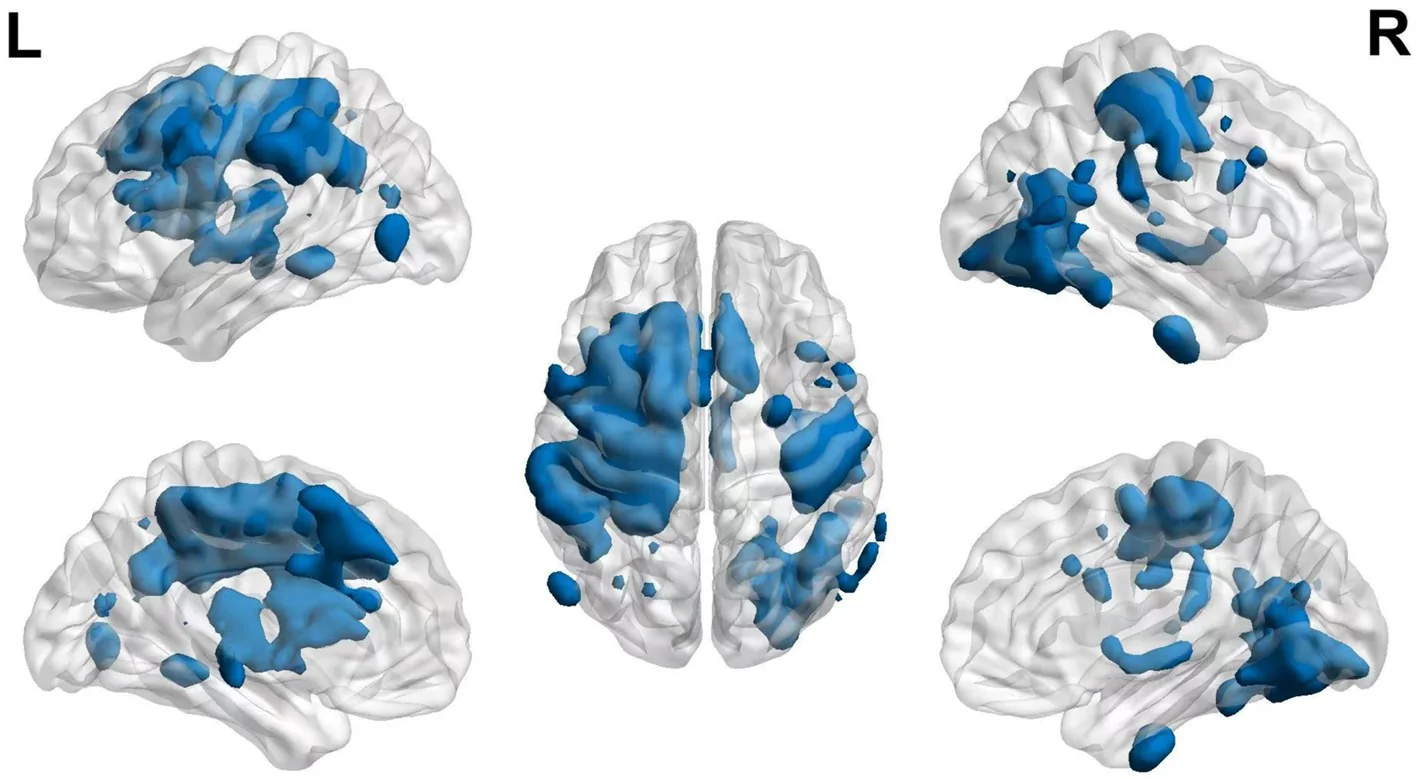
Changes in brain activity during taVNS with all three stimulation conditions in SCD.

Within the SCD group, one-way repeated-measures ANOVA demonstrated significant differences in ALFF values across the three stimulation conditions, with prominent effects observed in the bilateral frontal lobes, right temporal lobe, and right occipital lobe (FDR-corrected, *p* < 0.05) (Figure 3).

**Figure 4:**
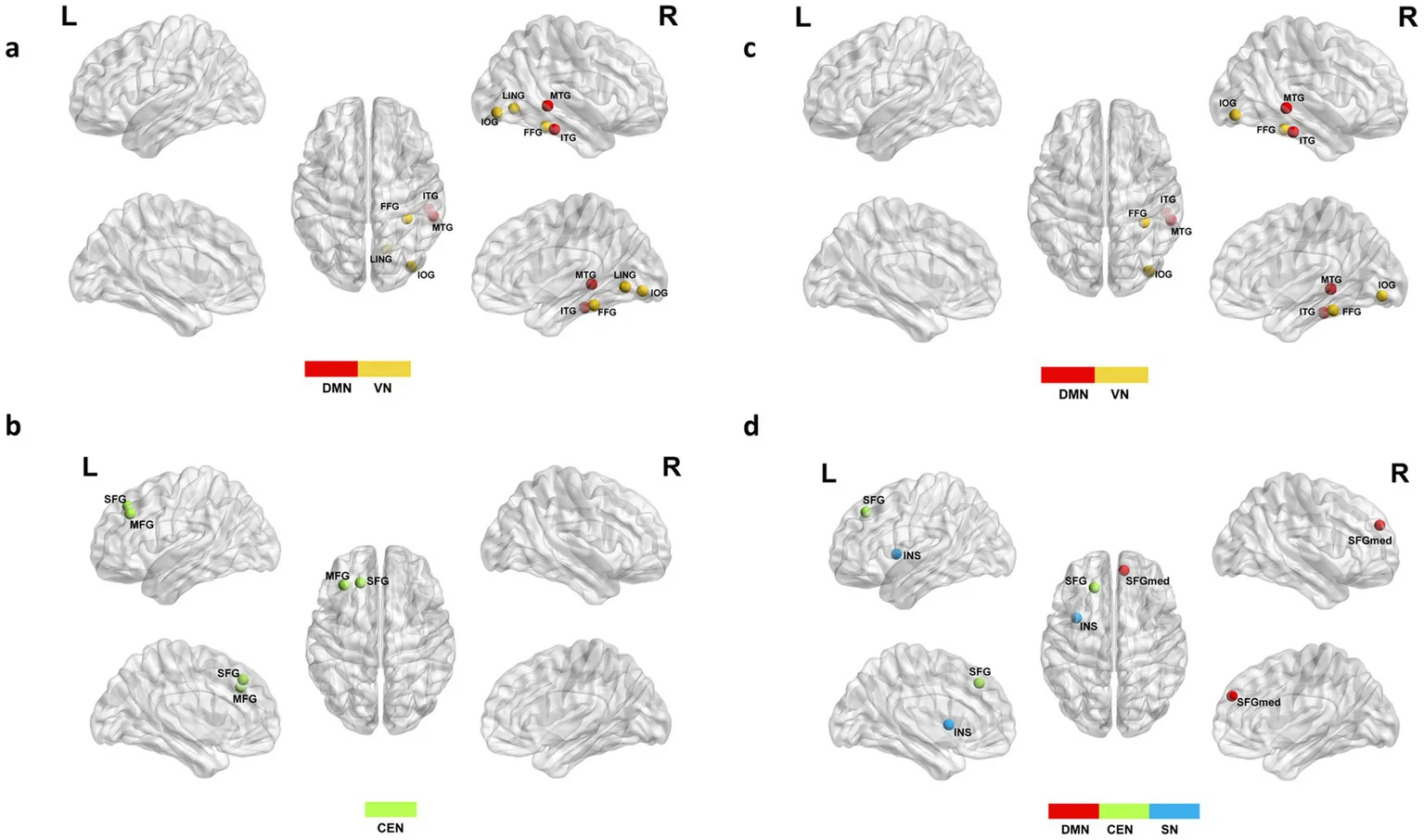
Changes in brain activity during 1 Hz and 20 Hz taVNS in individuals with SCD. Panels **(a,b)** show an increase and decrease in brain activity during 1 Hz taVNS, respectively; Panels **(c,d)** show an increase and decrease in brain activity during 20 Hz taVNS, respectively. FFG, fusiform gyrus; LING, lingual gyrus; IOG, inferior occipital gyrus; MTG, middle temporal gyrus; ITG, inferior temporal gyrus; MFG, middle frontal gyrus; SFG, superior frontal gyrus; INS, insula; SFGmed, medial superior frontal gyrus.

Compared with baseline in the SCD group, the ALFF values during 1 Hz taVNS were increased in the right fusiform gyrus (Fusiform_R), right lingual gyrus (Lingual_R), right inferior occipital gyrus (Occipital_Inf_R), right middle temporal gyrus (Temporal_Mid_R), and right inferior temporal gyrus (Temporal_Inf_R), and decreased in the left superior frontal gyrus (Frontal_Sup_L) and left middle frontal gyrus (Frontal_Mid_L) (Table 3; Figures 4a,b). During 20 Hz taVNS, the ALFF values were increased in the Temporal_Mid_R, Temporal_Inf_R, Occipital_Inf_R, and Fusiform_R, and decreased in the left insula (Insula_L), right medial superior frontal gyrus (Frontal_Sup_Med_R), and Frontal_Sup_L (Table 3; Figures 4c,d). No significant changes in brain activity were observed during StaVNS. No significant ALFF differences were observed between the 1 Hz and 20 Hz taVNS conditions (voxel level *p* < 0.001, cluster level *p* < 0.05, GRF correction, two-tailed).

**Table 3:** Brain activity changes in SCD patients during 1 Hz and 20 Hz taVNS.

	Brain regions	Cluster size	*t*-value	Peak MNI			ALFF value
				X	Y	Z	
1 Hz taVNS							
Cluster 1	Fusiform_R	50	5.2416	36	−69	−6	Increased
	Lingual_R	43					
	Occipital_Inf_R	34					
	Temporal_Mid_R	45					
	Temporal_Inf_R	14					
Cluster 2	Frontal_Mid_L	186	−5.0373	−45	0	30	Decreased
	Frontal_Sup_L	184					
20 Hz taVNS							
Cluster 1	Temporal_Mid_R	153	6.2586	39	−60	3	Increased
	Temporal_Inf_R	133					
	Occipital_Inf_R	108					
	Fusiform_R	57					
Cluster 2	Insula_L	34	−5.118	−36	0	18	Decreased
Cluster 3	Frontal_Sup_Med_R	32	−4.7828	6	18	45	Decreased
Cluster 4	Frontal_Sup_L	36	−4.992	−15	15	39	Decreased

SCD, subjective cognitive decline; MNI, Montreal Neurological Institute; X, Y, Z, indicate the coordinates according to the MNI. Statistical significance was defined as voxel-level *p* < 0.001, cluster-level *p* < 0.05, with GRF correction.

### 3.3 Correlation between ALFF values and neuropsychological performance

**Figure 5:**
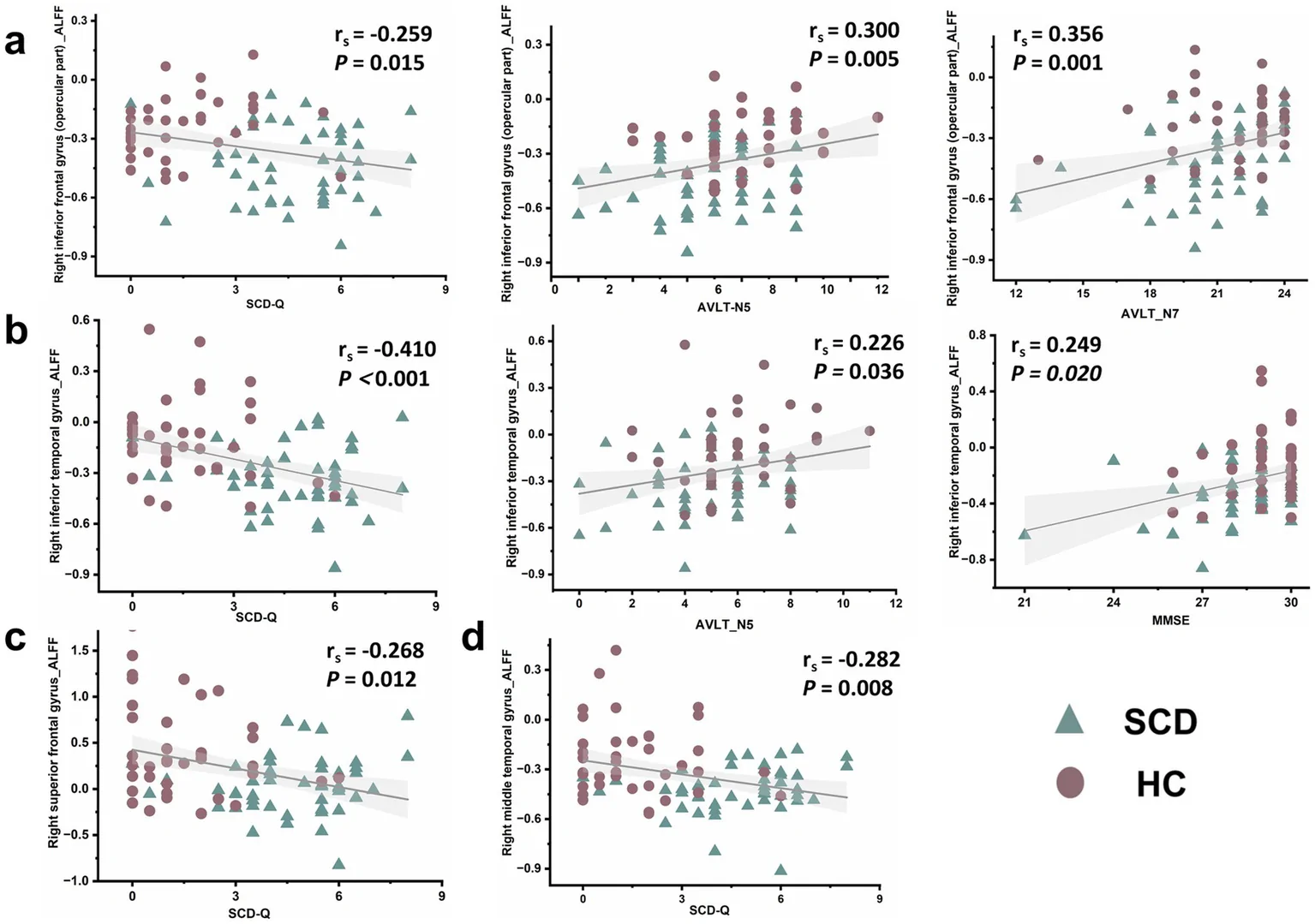
Correlations between ALFF values and neuropsychological scores in different brain regions. Panels **(a–d)** show correlations between ALFF values in the right inferior frontal gyrus (opercular part), right inferior temporal gyrus, right superior frontal gyrus, and right middle temporal gyrus and neuropsychological scores, respectively. Reported partial Spearman’s *rs* and *p* values were adjusted for sex. Scatterplots display raw data, and fitted lines represent unadjusted trends for visualization only.

Correlations between ALFF values and neuropsychological test scores are shown in Figure 5. ALFF values in the Frontal_Inf_Oper_R, Temporal_Inf_R, Temporal_Mid_R, and Frontal_Sup_R were negatively correlated with SCD-Q scores. ALFF values in the Frontal_Inf_Oper_R were positively correlated with delayed recall (AVLT-N5) and recognition (AVLT-N7) scores. ALFF values in the Temporal_Inf_R were positively correlated with delayed recall (AVLT-N5) and MMSE scores.

### 3.4 Exploratory ROI-based sensitivity analyses

Exploratory ROI based analyses showed a greater stimulation induced ALFF response in the Temporal_Inf_R during 20 Hz than during 1 Hz taVNS, uncorrected *p* = 0.024, Cohen’s *dz* = 0.33. However, the difference did not survive Bonferroni correction, adjusted *p* = 0.120. No significant differences were found in the remaining ROIs (Table 4).

**Table 4:** Exploratory ROI based comparisons of stimulation induced ALFF responses between 20 Hz and 1 Hz taVNS.

ROI	*t*	Uncorrected *p*	Adjusted *p*	Cohen’s *d*
Temporal_Mid_R	1.493	0.142	0.710	0.21
Temporal_Inf_R	2.332	0.024	0.120	0.33
Frontal_Sup_L	0.721	0.474	1.000	0.10
Frontal_Inf_Oper_R	0.340	0.736	1.000	0.05
Omnibus condition-effect ROI	0.442	0.661	1.000	0.06

The omnibus condition effect ROI was derived from a one way repeated measures ANOVA across resting state, 1 Hz taVNS, 20 Hz taVNS, and sham stimulation. Positive values indicate greater responses at 20 Hz. Bonferroni correction was applied across five ROIs.

## 4 Discussion

In this study, rs-fMRI was used to characterize the immediate neural responses to taVNS at different stimulation frequencies in individuals with SCD. At baseline, SCD was associated with reduced activity predominantly in the right hemisphere. Following stimulation, taVNS induced a pattern of increased spontaneous brain activity in the right hemisphere and decreased activity in the left hemisphere, primarily involving the default mode network (DMN), central executive network (CEN), and visual network (VN), and salience network (SN), ALFF changes observed under different stimulation frequency conditions involved partially different brain regions; however, direct comparisons between 1 Hz and 20 Hz stimulation conditions revealed no significant differences.

The right hemisphere aging model proposes that the right hemisphere exhibits greater age-related degeneration compared with the left hemisphere, a finding supported by recent evidence (Wei et al., 2022) and consistent with the right-lateralized patterns observed in aging and AD-related neurodegeneration. SCD has also been associated with hemispheric asymmetry in both structural and functional alterations. Structural MRI studies have shown that SCD is more likely to involve reduced right hippocampal volume (Yue et al., 2018). Consistent with this, our findings demonstrated reduced spontaneous neural activity in right hemisphere regions, including the Frontal_Sup, Frontal_Inf_Oper, Temporal_Mid, and Temporal_Inf.

Functionally, the Frontal_Sup is a key component of the CEN, which supports executive processes such as task execution and decision-making (Dosenbach et al., 2007). The inferior frontal and temporal regions are core nodes of the DMN, which is involved in internally directed cognition, including self-referential processing and emotional regulation, and plays a key role in large-scale network integration (Andrews-Hanna et al., 2010). Under normal conditions, the DMN and CEN exhibit an antagonistic relationship, with reciprocal modulation of activity (Raichle and Snyder, 2007). The concurrent reduction in activity observed in both networks suggests a dysfunction of coordinated network dynamics in SCD. Importantly, DMN is the most vulnerable brain network in the AD spectrum (Bai et al., 2008), with substantial overlap between its cortical hubs and regions of early amyloid-*β* deposition (Hoenig et al., 2018; Zott et al., 2018). The medial occipitotemporal cortex, along with the middle and inferior temporal gyri, may be among the earliest cortical regions affected in AD (Convit et al., 2000). Structural and functional alterations in these regions may therefore represent early markers of disease progression in cognitively-unimpaired individuals. Our findings support the notion that SCD represents a high-risk state for AD. We propose that right-lateralized reductions in spontaneous neural activity within key regions of the DMN and CEN may constitute a potential neurobiological mechanism underlying SCD.

Although individuals with SCD typically perform within the normal range on standardized neuropsychological tests, our results showed that the SCD group had significantly lower scores on the MMSE, AVLT, and SCD-Q compared with HCs. This finding is consistent with previous reports of subtle cognitive changes in SCD (Jessen et al., 2007; Tang et al., 2025). In the present study, ALFF values in regions showing abnormal activity may be associated with subjective cognitive complaints. Furthermore, ALFF values in the Frontal_Inf_Oper_R and Temporal_Inf_R were positively correlated with multiple AVLT subtests, which primarily assess episodic memory. Episodic memory impairment is a hallmark early feature of AD and may precede more global cognitive and functional decline. The inferior frontal gyrus is implicated in cognitive control, emotional processing, and working memory, while the temporal cortices, together with the hippocampal complex, is involved in episodic memory coding and retrieval (Liu et al., 2018; Cao et al., 2019). Given that episodic memory relies on distributed neural networks, reduced neural activity in the Frontal_Inf_Oper_R and Temporal_Inf_R may represent imaging characteristics associated with subjective cognitive complaints and episodic memory performance in SCD.

During 1 Hz-taVNS, increased ALFF values were observed mainly in regions within VN and DMN, whereas regions within CEN exhibited decreased activity, suggesting that the acute neural effects of taVNS extend beyond individual regions and involve alterations across large-scale functional networks. Notably, neural activity increased in the right middle and inferior temporal gyri, key nodes of the DMN, which also exhibited reduced activity at baseline. This pattern suggests that 1 Hz taVNS may influence network nodes exhibiting abnormal intrinsic activity in SCD and induce acute neural responses within these regions. The superior and middle frontal gyri, located in the dorsolateral prefrontal cortex (DLPFC), are core components of the CEN, responsible for controlling information encoding and memory retrieval. The left DLPFC is connected to multiple brain regions implicated in the pathophysiological progression of AD and is a common target for neuromodulation therapies such as transcranial magnetic stimulation (Alcalá-Lozano and Garza-Villarreal, 2018). The observed CEN-related activity changes may reflect the influence of taVNS on cognitive-related brain functional states in SCD. Alterations were also observed in the VN, which is primarily involved in visual information processing (Gerrits et al., 2019), and has additional roles in emotional regulation and social cognition (Purdy, 2011). Therefore, taVNS-induced VN alterations may reflect modulation of intrinsic activity in regions involved in sensory processing and cognition-related functions. Supporting this possibility, our previous research has shown that increased ALFF in the right fusiform gyrus is associated with cognitive improvement in patients with MCI following moxibustion treatment (Lai et al., 2022).

20 Hz taVNS elicited brain responses similar to those induced by 1 Hz taVNS, which may reflect the shared anatomic pathways of vagal afferent projections. Notably, reduced activity was observed in the left insula during 20 Hz taVNS. Insula is a part of the SN and is associated with emotional processing, attention, and interoception (Uddin, 2015). From the perspective of the triple-network model (Menon, 2011), SN serves a central switching function by detecting salient internal and external stimuli and dynamically coordinating activity between the DMN and CEN. Specifically, during stimulus-driven cognitive and emotional information processing, both CEN and SN typically exhibit increased activation, while DMN is typically suppressed during self-referential or low-demand tasks (Raichle et al., 2001; Greicius et al., 2003). Considering that SCD patients exhibited reduced intrinsic activity in DMN- and CEN-related regions at baseline, the insular activity changes induced by 20 Hz taVNS suggest that SN-related regions may be involved in the modulation of abnormal brain functional states by taVNS. However, given that this study was based on ALFF measurements, functional interactions and dynamic regulatory processes among different networks cannot be directly assessed. Furthermore, exploratory ROI analyses showed that the stimulation-induced ALFF response in the Temporal_Inf_R was greater following 20 Hz taVNS than 1 Hz taVNS (uncorrected *p* = 0.024). Although this finding suggests potential frequency sensitivity of some SCD-related abnormal regions, the absence of significance after Bonferroni correction prevents us from drawing conclusions regarding a stronger or frequency-specific neuromodulatory effect of 20 Hz taVNS.

Consistent with the neuromodulatory mechanisms reviewed in the Introduction, the widespread ALFF changes induced by taVNS likely arise from combined modulation of the LC–NE, cholinergic, dopaminergic and serotonergic systems. The LC–NE system, with its extensive cortical projections and role in regulating cortical excitability and large-scale network dynamics (Frangos et al., 2015), may provide a potential framework for interpreting the observed alterations involving the DMN, CEN, VN, and SN. Notably, taVNS was delivered to the left auricle in this study. The afferent vagal fibers from the left ear primarily transmit signals ipsilaterally to the nucleus tractus solitarius within the brainstem. However, the LC–NE system sends bilateral, broadly distributed cortical projections without obvious hemispheric preference. Therefore, the inherent anatomical characteristics of the LC–NE pathway cannot account for the prominent right-lateralized ALFF shifts observed here. Such rightward predominance is more likely attributed to intrinsic right frontotemporal abnormalities and heterogeneous regional brain sensitivity to vagal neuromodulation in SCD. The anatomical targets of thes cholinergic, dopaminergic and serotonergic systems overlap with multiple brain nodes showing significant ALFF shifts in our research. Despite these anatomical connections, ALFF only quantifies local spontaneous low-frequency neural oscillation. This imaging indicator lacks pathway specificity, so it is impossible to distinguish which neuromodulatory system dominates the detected brain activity changes.

In this study, no significant ALFF changes were observed during staVNS, which is inconsistent with some previous reports (Badran et al., 2018; Zhang et al., 2019). The possible reasons include: (i) stimulation at the control site, which is primarily innervated by the greater auricular nerve, may have been insufficient to elicit measurable central responses via its ascending projections; (ii) the insufficient sample size may have limited the statistical power to detect subtle effects.

This study has several limitations. First, the sample size was relatively small, which may reduce statistical stability and restrict generalizability. Second, this work only explored the instantaneous brain changes induced by single-session acute taVNS. Long-term repeated intervention and follow-up observation are lacking, so we cannot judge the persistent neuromodulatory effects. Third, we only collected resting-state fMRI data but did not conduct repeated neuropsychological assessment after stimulation. Without behavioral indicators, we cannot confirm whether brain activity changes translate to measurable cognitive improvements. Fourth, the enrolled subjects presented obvious sex imbalance, which may bring potential confounding effects on brain imaging results. Fifth, this trial adopted single-blind design, while we did not carry out post-experiment questionnaires to verify whether participants and operators successfully maintained blinding. Last, strict multiple-comparison correction was adopted to control false-positive results. This rigorous threshold inevitably led to relatively modest effect sizes, which may exclude subtle brain alterations with weak signals. Future studies will expand the sample volume, enroll gender-balanced cohorts, adopt long-term intervention protocols, match behavioral evaluations, complete blinding validity verification, and adopt hierarchical statistical strategies to optimize detection efficiency. In addition, the validated AHBA analytical approach can be combined with fMRI (Yan et al., 2024). This integrative strategy enables investigation into how taVNS-related gene expression changes correspond to brain functional remodeling, offering microscopic molecular clues to explain the macroscopic neural modulation observed in patients with SCD.

## 5 Conclusion

In conclusion, reduced spontaneous neural activity within right-lateralized DMN and CEN may represent a potential pathophysiological mechanism in SCD. Immediate taVNS, at both low and high frequencies modulated spontaneous activity across multiple cognition-related functional networks. These findings provide further insight into the acute neural effects of taVNS in SCD and support further investigation of its potential as a neuromodulatory strategy. Longitudinal and multimodal studies are warranted to evaluate the therapeutic potential and underlying mechanisms of taVNS in SCD.

## Data Availability

The raw data supporting the conclusions of this article will be made available by the authors, without undue reservation.

## References

[ref1] Alcalá-Lozano R. Garza-Villarreal E. A. (2018). Overlap of large-scale brain networks may explain the similar cognitive improvement of single-site vs multi-site rTMS in Alzheimer’s disease. Brain Stimul. 11, 942–944. doi: 10.1016/j.brs.2018.03.016, 29678442

[ref2] Andrews-Hanna J. R. Reidler J. S. Sepulcre J. Poulin R. Buckner R. L. (2010). Functional-anatomic fractionation of the brain’s default network. Neuron 65, 550–562. doi: 10.1016/j.neuron.2010.02.005, 20188659 PMC2848443

[ref3] Aston-Jones G. Cohen J. D. (2005). An integrative theory of locus coeruleus-norepinephrine function: adaptive gain and optimal performance. Annu. Rev. Neurosci. 28, 403–450. doi: 10.1146/annurev.neuro.28.061604.135709, 16022602

[ref4] Badran B. W. Dowdle L. T. Mithoefer O. J. LaBate N. T. Coatsworth J. Brown J. C. . (2018). Neurophysiologic effects of transcutaneous auricular vagus nerve stimulation (taVNS) via electrical stimulation of the tragus: a concurrent taVNS/fMRI study and review. Brain Stimul. 11, 492–500. doi: 10.1016/j.brs.2017.12.009, 29361441 PMC6487660

[ref5] Bai F. Zhang Z. Yu H. Shi Y. Yuan Y. Zhu W. . (2008). Default-mode network activity distinguishes amnestic type mild cognitive impairment from healthy aging: a combined structural and resting-state functional MRI study. Neurosci. Lett. 438, 111–115. doi: 10.1016/j.neulet.2008.04.021, 18455308

[ref6] Bauer S. Baier H. Baumgartner C. Bohlmann K. Fauser S. Graf W. . (2016). Transcutaneous Vagus nerve stimulation (tVNS) for treatment of drug-resistant epilepsy: a randomized, double-blind clinical trial (cMPsE02). Brain Stimul. 9, 356–363. doi: 10.1016/j.brs.2015.11.003, 27033012

[ref7] Cao Z. Bennett M. Orr C. Icke I. Banaschewski T. Barker G. J. . (2019). Mapping adolescent reward anticipation, receipt, and prediction error during the monetary incentive delay task. Hum. Brain Mapp. 40, 262–283. doi: 10.1002/hbm.24370, 30240509 PMC6865381

[ref8] Cao J. Zhang Y. Li H. Yan Z. Liu X. Hou X. . (2021). Different modulation effects of 1 Hz and 20 Hz transcutaneous auricular vagus nerve stimulation on the functional connectivity of the periaqueductal gray in patients with migraine. J. Transl. Med. 19:354. doi: 10.1186/s12967-021-03024-9, 34404427 PMC8371886

[ref9] Chen X. Lu B. Yan C.-G. (2018). Reproducibility of R-fMRI metrics on the impact of different strategies for multiple comparison correction and sample sizes. Hum. Brain Mapp. 39, 300–318. doi: 10.1002/hbm.23843, 29024299 PMC6866539

[ref10] Convit A. de Asis J. de Leon M. J. Tarshish C. Y. De Santi S. Rusinek H. (2000). Atrophy of the medial occipitotemporal, inferior, and middle temporal gyri in non-demented elderly predict decline to Alzheimer’s disease. Neurobiol. Aging 21, 19–26. doi: 10.1016/s0197-4580(99)00107-4, 10794844

[ref11] Dosenbach N. U. F. Fair D. A. Miezin F. M. Cohen A. L. Wenger K. K. Dosenbach R. A. T. . (2007). Distinct brain networks for adaptive and stable task control in humans. Proc. Natl. Acad. Sci. USA 104, 11073–11078. doi: 10.1073/pnas.0704320104, 17576922 PMC1904171

[ref12] Frangos E. Ellrich J. Komisaruk B. R. (2015). Non-invasive access to the Vagus nerve central projections via electrical stimulation of the external ear: fMRI evidence in humans. Brain Stimul. 8, 624–636. doi: 10.1016/j.brs.2014.11.018, 25573069 PMC4458242

[ref13] Gerges A. N. H. Williams E. E. R. Hillier S. Uy J. Hamilton T. Chamberlain S. . (2024). Clinical application of transcutaneous auricular vagus nerve stimulation: a scoping review. Disabil. Rehabil. 46, 5730–5760. doi: 10.1080/09638288.2024.2313123, 38362860

[ref14] Gerrits R. Van der Haegen L. Brysbaert M. Vingerhoets G. (2019). Laterality for recognizing written words and faces in the fusiform gyrus covaries with language dominance. Cortex 117, 196–204. doi: 10.1016/j.cortex.2019.03.010, 30986634

[ref15] Greicius M. D. Krasnow B. Reiss A. L. Menon V. (2003). Functional connectivity in the resting brain: a network analysis of the default mode hypothesis. Proc. Natl. Acad. Sci. USA 100, 253–258. doi: 10.1073/pnas.0135058100, 12506194 PMC140943

[ref16] Hoenig M. C. Bischof G. N. Seemiller J. Hammes J. Kukolja J. Onur Ö. A. . (2018). Networks of tau distribution in Alzheimer’s disease. Brain 141, 568–581. doi: 10.1093/brain/awx353, 29315361

[ref17] Jack C. R. Bennett D. A. Blennow K. Carrillo M. C. Dunn B. Haeberlein S. B. . (2018). NIA-AA research framework: toward a biological definition of Alzheimer’s disease. Alzheimers Dement. 14, 535–562. doi: 10.1016/j.jalz.2018.02.018, 29653606 PMC5958625

[ref18] Jessen F. Amariglio R. E. Buckley R. F. van der Flier W. M. Han Y. Molinuevo J. L. . (2020). The characterisation of subjective cognitive decline. Lancet Neurol. 19, 271–278. doi: 10.1016/S1474-4422(19)30368-0, 31958406 PMC7062546

[ref19] Jessen F. Amariglio R. E. van Boxtel M. Breteler M. Ceccaldi M. Chételat G. . (2014). A conceptual framework for research on subjective cognitive decline in preclinical Alzheimer’s disease. Alzheimers Dement. 10, 844–852. doi: 10.1016/j.jalz.2014.01.001, 24798886 PMC4317324

[ref20] Jessen F. Wiese B. Cvetanovska G. Fuchs A. Kaduszkiewicz H. Kölsch H. . (2007). Patterns of subjective memory impairment in the elderly: association with memory performance. Psychol. Med. 37, 1753–1762. doi: 10.1017/S0033291707001122, 17623488

[ref21] Jin S. Guo B. Hao W. Su X. Zhang T. Liu C. (2026). Neuromodulation techniques targeting neurotransmitter dysfunction: innovation in treatments for Alzheimer’s disease. Neural Regen. Res. 21, 4157–4168. doi: 10.4103/NRR.NRR-D-25-00582, 42148604 PMC13557669

[ref22] Kang S. Jeon S. Lee Y.-G. Ye B. S. (2023). Striatal dopamine transporter uptake, parkinsonism and cognition in Alzheimer’s disease. Eur. J. Neurol. 30, 3105–3113. doi: 10.1111/ene.15995, 37493955

[ref23] Klaassens B. L. van Gerven J. M. A. Klaassen E. S. van der Grond J. Rombouts S. A. R. B. (2019). Cholinergic and serotonergic modulation of resting state functional brain connectivity in Alzheimer’s disease. NeuroImage 199, 143–152. doi: 10.1016/j.neuroimage.2019.05.044, 31112788

[ref24] Lai Z. Zhang Q. Liang L. Wei Y. Duan G. Mai W. . (2022). Efficacy and mechanism of moxibustion treatment on mild cognitive impairment patients: an fMRI study using ALFF. Front. Mol. Neurosci. 15:852882. doi: 10.3389/fnmol.2022.852882, 35620445 PMC9127659

[ref25] Liu X. Chen W. Tu Y. Hou H. Huang X. Chen X. . (2018). The abnormal functional connectivity between the hypothalamus and the temporal gyrus underlying depression in Alzheimer’s disease patients. Front. Aging Neurosci. 10:37. doi: 10.3389/fnagi.2018.00037, 29487521 PMC5816744

[ref26] Ludwig M. Wienke C. Betts M. J. Zaehle T. Hämmerer D. (2021). Current challenges in reliably targeting the noradrenergic locus coeruleus using transcutaneous auricular vagus nerve stimulation (taVNS). Auton. Neurosci. 236:102900. doi: 10.1016/j.autneu.2021.102900, 34781120

[ref27] Menon V. (2011). Large-scale brain networks and psychopathology: a unifying triple network model. Trends Cogn. Sci. 15, 483–506. doi: 10.1016/j.tics.2011.08.003, 21908230

[ref28] Merrill C. A. Jonsson M. A. G. Minthon L. Ejnell H. C-son Silander H. Blennow K. . (2006). Vagus nerve stimulation in patients with Alzheimer’s disease: additional follow-up results of a pilot study through 1 year. J. Clin. Psychiatry 67, 1171–1178. doi: 10.4088/jcp.v67n0801, 16965193

[ref29] Murphy A. J. O’Neal A. G. Cohen R. A. Lamb D. G. Porges E. C. Bottari S. A. . (2023). The effects of transcutaneous vagus nerve stimulation on functional connectivity within semantic and hippocampal networks in mild cognitive impairment. Neurotherapeutics 20, 419–430. doi: 10.1007/s13311-022-01318-4 36477709 PMC10121945

[ref30] Mwamburi M. Liebler E. J. Tenaglia A. T. (2017). Review of non-invasive vagus nerve stimulation (gammaCore): efficacy, safety, potential impact on comorbidities, and economic burden for episodic and chronic cluster headache. Am. J. Manag. Care 23, S317–S325. 29144717

[ref31] Peuker E. T. Filler T. J. (2002). The nerve supply of the human auricle. Clin. Anat. 15, 35–37. doi: 10.1002/ca.1089, 11835542

[ref32] Purdy R. A. (2011). The role of the visual system in migraine: an update. Neurol. Sci. 32, 89–93. doi: 10.1007/s10072-011-0541-4, 21533721

[ref33] Raichle M. E. MacLeod A. M. Snyder A. Z. Powers W. J. Gusnard D. A. Shulman G. L. (2001). A default mode of brain function. Proc. Natl. Acad. Sci. USA 98, 676–682. doi: 10.1073/pnas.98.2.676, 11209064 PMC14647

[ref34] Raichle M. E. Snyder A. Z. (2007). A default mode of brain function: a brief history of an evolving idea. NeuroImage 37, 1083–1090. doi: 10.1016/j.neuroimage.2007.02.041, 17719799

[ref35] Ren R. Qi J. Lin S. Liu X. Yin P. Wang Z. . (2022). The China Alzheimer report 2022. Gen. Psychiatr. 35:e100751. doi: 10.1136/gpsych-2022-100751, 35372787 PMC8919463

[ref36] Sjögren M. J. C. Hellström P. T. O. Jonsson M. A. G. Runnerstam M. Silander H. C.-S. Ben-Menachem E. (2002). Cognition-enhancing effect of vagus nerve stimulation in patients with Alzheimer’s disease: a pilot study. J. Clin. Psychiatry 63, 972–980. doi: 10.4088/jcp.v63n1103, 12444809

[ref37] Slot R. E. R. Sikkes S. A. M. Berkhof J. Brodaty H. Buckley R. Cavedo E. . (2019). Subjective cognitive decline and rates of incident Alzheimer’s disease and non-Alzheimer’s disease dementia. Alzheimers Dement. 15, 465–476. doi: 10.1016/j.jalz.2018.10.003, 30555032 PMC6465066

[ref38] Tang W. Zeng Q. Xie K. Cui X. Jin X. Han Y. . (2025). Disrupted brain networks underlying high-fidelity memory retrieval in subjective cognitive decline: a task-based fMRI study. Alzheimers Dement. 21:e14431. doi: 10.1002/alz.14431, 39732511 PMC11848185

[ref39] Uddin L. Q. (2015). Salience processing and insular cortical function and dysfunction. Nat. Rev. Neurosci. 16, 55–61. doi: 10.1038/nrn3857, 25406711

[ref40] Urbin M. A. Lafe C. W. Simpson T. W. Wittenberg G. F. Chandrasekaran B. Weber D. J. (2021). Electrical stimulation of the external ear acutely activates noradrenergic mechanisms in humans. Brain Stimul. 14, 990–1001. doi: 10.1016/j.brs.2021.06.002, 34154980

[ref41] Walsh S. Merrick R. Milne R. Nurock S. Richard E. Brayne C. (2024). Considering challenges for the new Alzheimer’s drugs: clinical, population, and health system perspectives. Alzheimers Dement. 20, 6639–6646. doi: 10.1002/alz.14108, 39105453 PMC11497759

[ref42] Wang L. Zhang J. Guo C. He J. Zhang S. Wang Y. . (2022). The efficacy and safety of transcutaneous auricular vagus nerve stimulation in patients with mild cognitive impairment: a double blinded randomized clinical trial. Brain Stimul. 15, 1405–1414. doi: 10.1016/j.brs.2022.09.003, 36150665

[ref43] Wei Y.-C. Kung Y.-C. Huang W.-Y. Lin C. Chen Y.-L. Chen C.-K. . (2022). Functional connectivity dynamics altered of the resting brain in subjective cognitive decline. Front. Aging Neurosci. 14:817137. doi: 10.3389/fnagi.2022.817137, 35813944 PMC9263398

[ref44] Yan T.-C. Yu S.-W. Wang X.-Y. Ren L. Li D. Chu W.-Y. . (2024). Allen human brain atlas and magnetic resonance imaging in schizophrenia. Meta-Radiology 2:100087. doi: 10.1016/j.metrad.2024.100087

[ref45] Yuan H. Silberstein S. D. (2016). Vagus nerve and vagus nerve stimulation, a comprehensive review: part I. Headache 56, 71–78. doi: 10.1111/head.12647, 26364692

[ref46] Yue L. Wang T. Wang J. Li G. Wang J. Li X. . (2018). Asymmetry of Hippocampus and amygdala defect in subjective cognitive decline among the community dwelling Chinese. Front. Psych. 9:226. doi: 10.3389/fpsyt.2018.00226, 29942265 PMC6004397

[ref47] Zang Y.-F. He Y. Zhu C.-Z. Cao Q.-J. Sui M.-Q. Liang M. . (2007). Altered baseline brain activity in children with ADHD revealed by resting-state functional MRI. Brain Dev. 29, 83–91. doi: 10.1016/j.braindev.2006.07.002, 16919409

[ref48] Zhang H. Lin S.-C. Nicolelis M. A. L. (2010). Spatiotemporal coupling between hippocampal acetylcholine release and theta oscillations in vivo. J. Neurosci. 30, 13431–13440. doi: 10.1523/JNEUROSCI.1144-10.2010, 20926669 PMC2988451

[ref49] Zhang Y. Liu J. Li H. Yan Z. Liu X. Cao J. . (2019). Transcutaneous auricular vagus nerve stimulation at 1 Hz modulates locus coeruleus activity and resting state functional connectivity in patients with migraine: an fMRI study. Neuroimage Clin. 24:101971. doi: 10.1016/j.nicl.2019.101971, 31648171 PMC7239932

[ref50] Zhang D. Raichle M. E. (2010). Disease and the brain’s dark energy. Nat. Rev. Neurol. 6, 15–28. doi: 10.1038/nrneurol.2009.198, 20057496

[ref51] Zott B. Busche M. A. Sperling R. A. Konnerth A. (2018). What happens with the circuit in Alzheimer’s disease in mice and humans? Annu. Rev. Neurosci. 41, 277–297. doi: 10.1146/annurev-neuro-080317-061725, 29986165 PMC6571139

